# Malaria outbreak facilitated by increased mosquito breeding sites near houses and cessation of indoor residual spraying, Kole district, Uganda, January-June 2019

**DOI:** 10.1186/s12889-022-14245-y

**Published:** 2022-10-12

**Authors:** Maureen Nabatanzi, Vivian Ntono, John Kamulegeya, Benon Kwesiga, Lilian Bulage, Bernard Lubwama, Alex. R. Ario, Julie Harris

**Affiliations:** 1 Uganda Public Health Fellowship Program, Ministry of Health, Kampala, Uganda; 2 Integrated Epidemiology, Surveillance and Public Health Emergencies Department, Ministry of Health, Kampala, Uganda; 3 US Centers for Disease Control and Prevention, Kampala, Uganda

**Keywords:** Malaria, Outbreak, Stagnant water, Uganda, IRS

## Abstract

**Background:**

In June 2019, surveillance data from the Uganda’s District Health Information System revealed an outbreak of malaria in Kole District. Analysis revealed that cases had exceeded the outbreak threshold from January 2019. The Ministry of Health deployed our team to investigate the areas and people affected, identify risk factors for disease transmission, and recommend control and prevention measures.

**Methods:**

We conducted an outbreak investigation involving a matched case-control study. We defined a confirmed case as a positive malaria test in a resident of Aboke, Akalo, Alito, and Bala sub-counties of Kole District January–June 2019. We identified cases by reviewing outpatient health records. Exposures were assessed in a 1:1 matched case-control study (n = 282) in Aboke sub-county. We selected cases systematically from 10 villages using probability proportionate to size and identified age- and village-matched controls. We conducted entomological and environmental assessments to identify mosquito breeding sites. We plotted epidemic curves and overlaid rainfall, and indoor residual spraying (IRS). Case-control exposures were combined into: breeding site near house, proximity to swamp and breeding site, and proximity to swamp; these were compared to no exposure in a logistic regression analysis.

**Results:**

Of 18,737 confirmed case-patients (AR = 68/1,000), Aboke sub-county residents (AR = 180/1,000), children < 5 years (AR = 94/1,000), and females (AR = 90/1,000) were most affected. Longitudinal analysis of surveillance data showed decline in cases after an IRS campaign in 2017 but an increase after IRS cessation in 2018–2019. Overlay of rainfall and case data showed two malaria upsurges during 2019, occurring 35–42 days after rainfall increases. Among 141 case-patients and 141 controls, the combination of having mosquito breeding sites near the house and proximity to swamps increased the odds of malaria 6-fold (OR = 6.6, 95% CI = 2.24–19.7) compared to no exposures. Among 84 abandoned containers found near case-patients’ and controls’ houses, 14 (17%) had mosquito larvae. Adult Anopheles mosquitoes, larvae, pupae, and pupal exuviae were identified near affected houses.

**Conclusion:**

Stagnant water formed by increased rainfall likely provided increased breeding sites that drove this outbreak. Cessation of IRS preceded the malaria upsurges. We recommend re-introduction of IRS and removal of mosquito breeding sites in Kole District.

## Background

Malaria is transmitted to humans when they are bitten by infective female Anopheles mosquitoes with Plasmodium parasite sporozoites in the salivary glands. Malaria is endemic in Uganda; 90–95% of the country has stable malaria transmission [1]. Anopheles gambiae and Anopheles funestus species, which are endophagic and endophilic (bite and rest indoors), are the most common malaria vectors in Uganda [1]. Malaria transmission intensity partly depends on the vector density, which is in turn dependent on favorable temperatures and the presence of mosquito breeding sites. In Uganda, transmission is ongoing throughout the year, with two annual peaks that typically follow the two rainy seasons in March–May and August–October [2].

Uganda has reported multiple, geographically diverse malaria outbreaks over the last 20 years [3–6]. In 2017, nearly 20% of Ugandans suffered at least one episode of malaria, and malaria was responsible for 5% of all deaths in the country [7]. Despite a 52% decline in national malaria-related deaths between 2016/2017 and 2017/2018, malaria prevalence was 9% among children under 5 in 2018/2019 financial year [8, 9]. Through the Uganda Malaria Reduction Strategic Plan (UMRSP) 2014–2020, the Ministry of Health (MoH) implemented activities to reduce annual malaria morbidity, mortality, and parasite prevalence. This involved case and fever management, referral, provision of essential diagnostics and antimalarials, behavioral change communication and technical support to affected districts. Long-lasting insecticide-treated nets (LLINs) were distributed continuously through antenatal and immunization clinics and nationally every three years, and indoor residual spraying (IRS) was conducted annually in selected districts to control vectors [10].

Kole District (altitude: 1,150 m above sea level) is located in Lango sub-region of northern Uganda and has a population of approximately 280,000 persons [11]. It has two seasonal rainfall peaks in March to May and September to November, with annual rainfall ranging from 875 mm to 1,500 mm. As of 2019, Kole District had 16 health facilities, including one Health Centre (HC) IV, five HC IIIs, six HC IIs and four clinics. All have capacity to test for and treat malaria.

Ten districts in Eastern and mid-Northern Uganda, including Kole, received IRS annually during 2009–2014, which contributed to reducing the malaria burden. However, during 2014–2016, IRS support shifted to other districts, leading to increases in malaria occurrence in the former 10 districts in Eastern and mid-Northern Uganda. As an intervention to address this resurgence, single round of IRS was conducted in 2017 [8]. Since that time, no additional IRS campaigns have been carried out in the area, and Kole, like other districts in Northern Uganda, continues to experience seasonal malaria outbreaks [3].

In June 2019, routine analysis of malaria surveillance data from Uganda’s District Health Information System (DHIS2) showed a malaria outbreak in Kole District. We plotted a malaria normal channel graph, a plot of weekly confirmed malaria cases in Kole District over the previous five years (2013–2018) analyzed into upper (75th percentile) and lower (25th percentile) epidemic thresholds of expected cases, and compared to 2019 cases [12]. Starting in January to June 2019, malaria cases exceeded the upper epidemic threshold. Further disaggregation of the data showed four sub-counties were the most highly affected. The MoH deployed a study team composed of national rapid response members, district and community health workers to respond to this outbreak. The team investigated to determine the extent, identify risk factors for increased transmission in Kole District, and to recommend control and prevention measures.

## Methods

### Outbreak area

We extracted malaria surveillance data for Kole District from the District Health Information System (DHIS2). We computed malaria cases by sub-county and drew malaria channel graphs to identify sub-counties with the highest burden of cases during the outbreak period. The four most-affected sub-counties: Aboke, Akalo, Alito, and Bala were selected for the investigation of the outbreak. In the sub-counties, we purposively selected and visited Aboke HC IV, Akole HC III, Apalabarowoo HC III, Bala HC III and Opeta HC III. During our investigation, the district health team informed us of antimalaria stockouts at lower-level public health facilities, which led to referral of cases to these five health facilities.

### Case definition and finding

We defined a confirmed case as a positive malaria result by the histidine-rich protein II rapid diagnostic test (mRDT) or microscopy in a resident of the four most-affected sub-counties (Aboke, Akalo, Alito, and Bala sub-counties) from 1 January to 30 June 2019. We purposively reviewed outpatient health records in five higher-level health facilities (1 HC IV and 4 HC IIIs) to search for confirmed malaria cases in these sub-counties. This purposive selection was based on information by the district health team that antimalaria stockouts at lower-level public health facilities had led to referral of cases to these five. Using the out-patient records, we line-listed all case-patients who fit the case definition. For each case-patient, we abstracted information on confirmatory diagnostic test done, age, sex, village, parish, and sub-county of residence.

### Descriptive epidemiology

Using the line list, we described case-patients by person, place, and time. We defined attack rate as the number of malaria cases during January to June 2019 divided by the population at risk. Populations at risk used were extracted from the 2019 Uganda National Population Census projections for Kole District [11]. Consequently, we computed attack rates by age-group, sex, sub-county, parish and village; groups with the highest attack rates were classified as the most affected. We drew a map of the district indicating affected sub-counties. Rainfall data for Kole District for January to June 2019 were abstracted from the online weather resource AccuWeather Inc. [13]. An epidemic curve was drawn to describe the distribution of malaria cases in the district during January to June 2019 and. rainfall data superimposed over the curve. Another epidemic curve of malaria cases in Kole District from 2016 to 2019 was drawn with data on IRS obtained from MoH records [8] superimposed over the cases. Using surveillance data from the DHIS2, we plotted a graph showing trends in confirmed malaria cases in Kole and included IRS interventions in the district from 2016 to 2019.

### Environmental assessment

In Aboke sub-county, we selected Ogwangacuma Parish which had the highest attack rate (345 per 1,000) and in turn selected Aweingwec Village that reported the highest number of malaria cases (n = 2,392) during January – June 2019. In Aweingwec Village, we conducted transect walks by systematically walking with community health workers to explore the environment for stagnant water, swamps and potential risk factors for mosquito breeding and malaria transmission. We identified active and potential breeding sites for mosquitoes near houses and the environment.

### Entomological assessment

In Aboke sub-county, we selected Akwirididi parish, one of the two most affected parishes, to conduct entomological assessments. In 2019, Akwirididi had 28 villages and 2,748 households, from which we selected a random sample of 20 houses to assess the mosquito density. In each house, we used the pyrethrum spray catch method to collect indoor resting mosquitoes by spraying pyrethrum insecticide inside the house and collecting mosquitoes that were knocked down on a white sheet laid on the ground. We conducted daily pyrethrum spray catches from 6 to 10 am during the 13–15 July 2019. The dead mosquitoes were collected using forceps, packed in petri dishes, and transported to the laboratory for counting and identification [14]. The mosquito indoor resting density (IRD) was computed using the formula:

$${\text{IRD}}=\frac{{\left( {{\text{no}}{\text{.}}\;of\;{\text{mosquitoes collected indoors}} \div {\text{no}}{\text{.of houses}}} \right)}}{{{\text{number of mornings}}}}$$

At breeding sites around the sampled houses, scoops were used to collect larvae, pupae, and pupal exuviae; strainers and filter cloths were used to remove excess water. Residual material was then transported to the laboratory for counting and identification.

### Hypothesis generation interviews

In Aboke, the most affected sub-county, we purposively selected Ogwangacuma parish because it had the highest attack rate. In this parish, we conveniently sampled 20 case-patients. The community health workers on our team introduced the purpose of the investigation and supported translation from the local language when necessary. Case-patients were interviewed about possible behavioral and environmental exposures associated with malaria transmission; we also observed their environment for potential risk factors. The exposure variables explored included living close to swampy areas, human activities in and around swamps, presence of stagnant water near houses following rainfall (present during our visits), and LLIN use during the two weeks before symptom onset.

### Case-control study

We conducted a case-control study to test the generated hypotheses in two parishes of Aboke sub-county. The parishes of Ogwangacuma and Akwirididi were selected because of their high attack rates. From these two parishes, we further selected the ten most affected villages. The number of cases and controls selected from each affected village was estimated using the probability proportionate to size sampling method where each village contributed persons proportional to the village’s attack rate [3].

We defined a case-patient as a resident of Ogwangacuma or Akwirididi Parish in Aboke sub-county with evidence of a positive malaria RDT in the previous four weeks (8 June to 8 July 2019). For each case-patient, evidence of malaria RDT was abstracted from health facility out-patient records. We defined a control as a resident of Ogwangacuma or Akwirididi Parish with no signs or symptoms of malaria and no positive test for malaria in the same previous four weeks. Cases and controls were matched by village of residence and age (within 5 years). We used a case to control ratio of 1:1, selecting 141 cases and 141 controls (n = 282).

We used systematic sampling to select cases and controls. A list of all houses per village was obtained from the respective local council leaders and used as the sampling frame from which we calculated the sampling interval. All houses in the sampling frame were assigned a number and OpenEpi™ was used to generate one random number which served as the starting point for selecting the first house from which to select a case-patient. After this, we used the sampling interval to select the remaining cases. The remaining houses were assigned numbers and random numbers generated in OpenEpi™ and used to select matching controls. If the house had a case-patient or didn’t have an age-matched person, it was replaced by a neighboring house. We administered a questionnaire to each case-patient with questions on demographics and exposure to malaria risk factors during the two weeks before symptom onset. The same questionnaire was administered to controls to assess exposure to malaria risk factors during the two weeks before their matched case-patient’s symptom onset. For case-patients or controls who were minors, the questionnaire was administered to guardians. At selected houses, we looked out for abandoned containers with stagnant water and visible mosquito larvae. Any vessel found in the open around the house but no longer in use that could store an amount of water to allow mosquitoes to lay their eggs was considered as an abandoned container.

### Data management and analysis

Data were first entered, cleaned in Microsoft Excel before being imported into Epi Info 7.2 to generate descriptive statistics. In Epi Info, we analyzed the case-control data by creating the following combined exposure categories: [1] Breeding site near house (a combination of either abandoned containers or stagnant water near house), [2] Proximity to swamp (a combination of either house < 500 m of swamp exposures or farm < 500 m of swamp exposures), [3] Combination of breeding site near house and proximity to swamp, and [4] Common reference category (no breeding site near house and no proximity to swamp). This enabled us to compare the individual effect of each of the combined exposures (categories 1 and 2), and the joint effect of all the exposures (category 3) to a common reference of no exposures (category 4). Using logistic regression analysis, we computed odds ratios (OR) and their 95% confidence intervals.

## Results

### Descriptive epidemiology

We line-listed 18,737 confirmed case-patients in the four most affected sub-counties of Kole District (Aboke, Akalo, Alito, and Bala). The overall attack rate (AR) was 68/1,000 with no deaths. The median age was 12 years (range: <1 to 98 years). Children under 5 years were the most affected (AR = 94/1,000) followed by children aged 5–18 years (71/1,000). Females (AR = 90/1,000) were more affected than the males (AR = 45/1,000) (Table 1).

**Table 1:** Attack rates by sex and age-group during a malaria outbreak in Kole District, Uganda, January-June 2019

Characteristics		Population	Cases	% of Cases (n = 18,737)	Attack Rate/1,000
**Sex**	Female	139,700	12,535	67	90
	Male	136,600	6,202	33	45
**Age (years)**	Children under 5	48,380	4,419	24	91
	Children 5-18y	116,240	7,744	41	67
	Adult > 18y	111,680	6,574	35	59

**Fig. 1:**
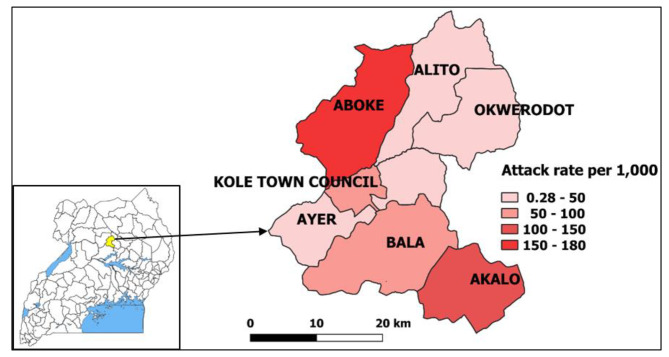
Map of affected sub-counties during a malaria outbreak in Kole District, Northern Uganda, January-June 2019. Inset: location of Kole District in Uganda. (*Note.* Results are presented for 7 sub-counties instead of the 4 visited due to the referrals from health facilities located in other sub-counties during the period considered for the investigation. The outpatient department register collects data on village, parish, and sub-county of residence of the case-patients.)

Of the four sub-counties visited, Aboke had a higher attack rate (AR = 180/1,000) in comparison to Alito, Akalo and Bala (Fig. 1).

**Fig. 2:**
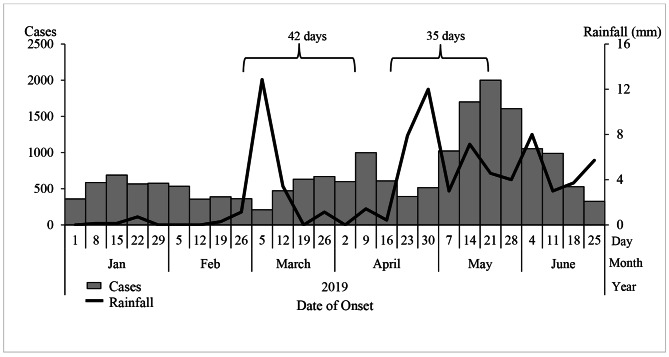
Weekly confirmed cases (red bars) and weekly rainfall (blue line) during a malaria outbreak in Kole District, Uganda, January-June 2019

The epidemic curve showed peaks in malaria cases on 9 April and 21 May 2019. The peaks in malaria cases followed increases in rainfall by 35-42-day intervals. We also observed peak-to-peak increases; May’s peak was the highest following the second increase in rainfall (Fig. 2).

**Fig. 3:**
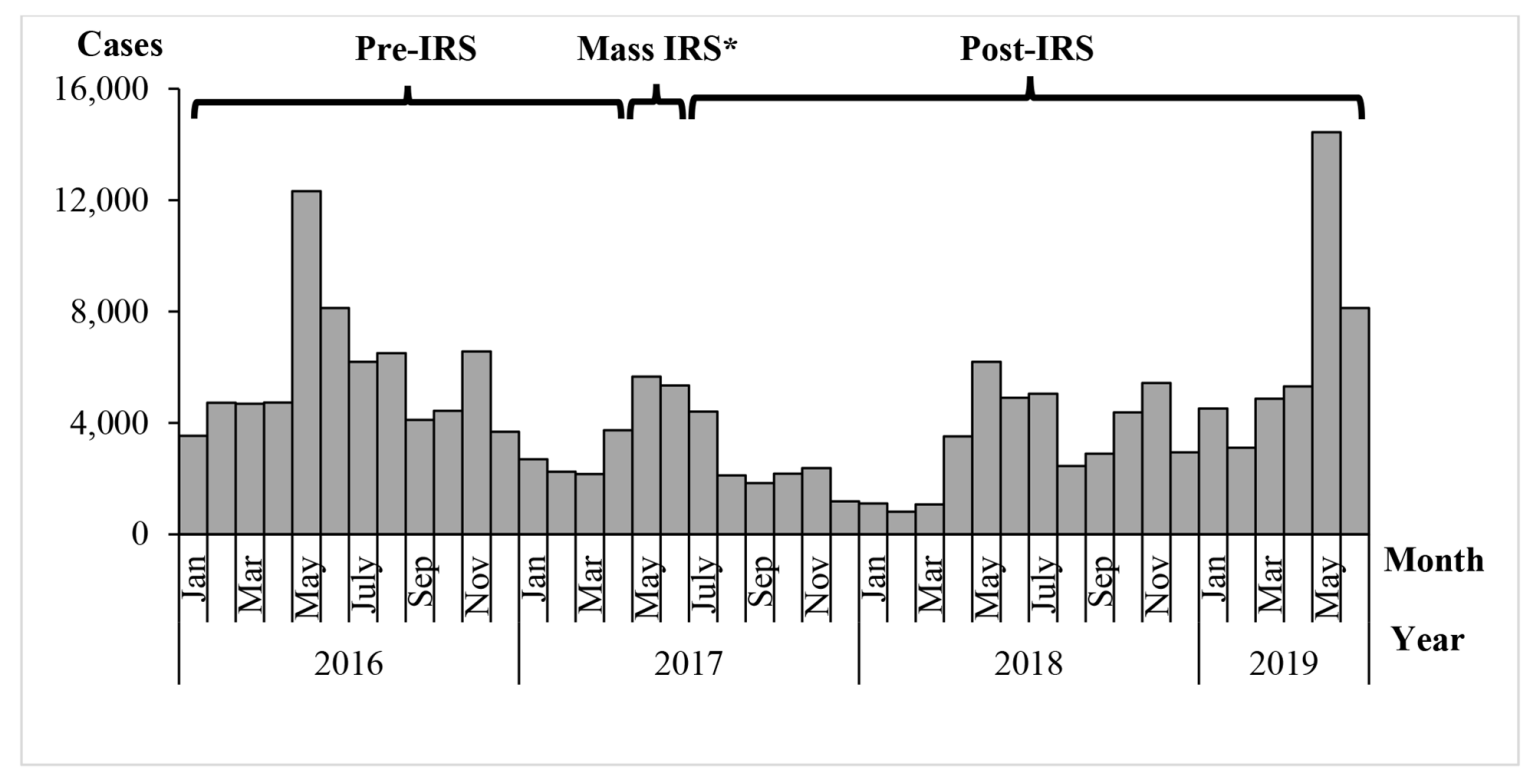
Monthly confirmed malaria cases and timing of mass indoor residual spraying in Kole District, Uganda, 2016–2019. Note*: In addition to the mass IRS, an LLIN distribution campaign was conducted

A graph of confirmed malaria cases in Kole District from 2016 to 2019 showed annual seasonal peaks in malaria cases during May-July and October-November (Fig. 3). During May and June 2017, Kole District conducted a mass indoor residual spraying (IRS) campaign, which appeared to reduce cases over the following year. Monthly cases in 2019 were high in comparison to 2016, 2017 and 2018.

### Entomological assessment findings

Around the 20 houses we visited, we identified any stagnant water containing areas or containers with mosquito larvae as sites for breeding. From these 20 houses, 262 adult Anopheles mosquitoes were identified during knockdown. Of the 262 adult mosquitoes, 204 (78%) were female, of whom 140 (69%) were Anopheles gambiae and 64 (31%) were Anopheles funestus. Among these, 171 (84%) were freshly fed. The average indoor resting density of malaria vectors was 4 mosquitoes per house per night. In stagnant water near the 20 houses, we identified an average of 10 Anopheles larvae, four Anopheles pupae, and multiple Anopheles exuviae per 500ml scoop; these were of gambiae and fenustus species.

### Environmental assessment findings

In Aboke sub-county, the main economic activity was subsistence farming. On rice farms in swampy areas, we identified stagnant water with visible Anopheles mosquito larvae. We also identified man-made ponds being used for fish farming. These were surrounded by ditches which had filled with rainwater that had stagnated; we found Anopheles mosquito larvae in the ditches.

### Hypothesis generation findings

Among the 20 case-patients interviewed, 17 (85%) lived within 500 m of a swamp, 15 (75%) farmed within 500 m of a swamp and 11 (55%) had stagnant water near their house. Based on the descriptive epidemiology, environmental and entomological assessments, and interview findings, the study team hypothesized that stagnation of rain water in swampy areas, ditches, and around houses favored mosquito breeding.

### Case-control study findings

Among 141 case-patients and 141 controls, having breeding sites near the house either as abandoned containers or as stagnant water (OR = 1.09, 95% CI = 0.24–5.02) was not associated with malaria infection. Proximity to swamps either as farm or house less than 500 m to the swamp (OR = 1.05, 95% CI = 0.45–2.4) was also not associated with malaria infection. Further analysis of the risky exposures in combination revealed a possible combined effect. The combination of having breeding sites near house and proximity to swamps increased the odds of malaria 6-fold (OR = 6.6, 95% CI = 2.24–19.7) (Table 2). We identified a total of 84 abandoned containers near participants’ houses, 14 (17%) of which had visible mosquito larvae. Examples of abandoned containers identified included old jerry cans, saucepans and basins.

Of the 282 study participants, 227 (80%) reported using an LLIN the previous night that is, 80% (113/141) of case-patients compared to 81% (114/141) controls.

**Table 2:** Distribution of exposure status among case-patients and controls during a malaria outbreak in Kole District, Uganda, January-June 2019

Exposure	n (%) cases exposed	n (%) controls exposed	Total exposed	Odds Ratio	95% CI
Reference (No breeding site near house and no proximity to swamp)	11 (7.8)	15 (11)	26	Ref	Ref
Breeding site (Stagnant water or abandoned containers) near house	4 (2.8)	5 (3.5)	9	1.09	0.24–5.02
Proximity to swamp (House or farm < 500 m from swamp)	87 (62)	113 (80)	247	1.05	0.45–2.4
Combination of Breeding site near house and Proximity to swamp	39 (28)	8 (5.7)	47	6.6	2.24–19.7
Total	141	141	282	-	-

*All exposures are compared with the no-exposure reference group

CI: confidence interval.

## Discussion

There was in increase in malaria cases in Kole District in 2019. While IRS in 2017 appeared to reduce the malaria levels in 2017 and early 2018, its effect appeared to have worn off by 2019. Peaks in malaria cases followed rains in 2019. Persons living in Aboke sub-county, children under five years, and women were the more affected by this outbreak in comparison to other groups. There were many freshly-fed adult female mosquitoes in houses in the affected area, implying that residents were being actively bitten even during our investigation period, which occurred after the peak of cases. Risky exposures associated with malaria included having abandoned containers and stagnant water near work or house.

In Uganda, the main malaria control measures are IRS, distribution of LLINs, accurate diagnosis and prompt treatment, and intermittent preventive treatment of pregnant women [10]. In 2017, the MoH conducted a routine LLIN distribution that achieved 88% national coverage [8], which, complemented by the mass IRS, should have been sufficient to have a protective effect. However, high LLIN coverage rates don’t always reflect use; the 2018/2019 Uganda Malaria Indicator Survey reported national net use of 59% [9]. Although reported net use in our study was 80%,in areas with favorable vector and rainfall conditions, regular LLIN use should be combined with other interventions such as IRS to reduce the mosquito population sufficiently to impact malaria infection rates [4]. However, the expense of IRS often precludes its regular application or universal coverage.

We noted increases in malaria peaks approximately 5–6 weeks after rainfall peaks. This is a well-described phenomenon in the malaria literature and has been reported previously [3, 4]. This first increase in rainfall, during early March of 2019 could have facilitated an increase in mosquito breeding sites. Successive peaks in rainfall could have favored three mosquito breeding cycles of two weeks each, resulting in a generational increase in mosquito density[15]. However, rainfall itself is not enough to guarantee mosquito breeding. Opportunities for breeding sites exist when there is stagnant water and flooding near places where people live, work or rest [3, 4, 16]. Farming in swamps can also modify the water temperature, resulting in favorable conditions for mosquito breeding [17]. We found pools of water in open abandoned containers surrounding houses, as well as stagnant water near houses resulting from flooding which served as sites for mosquitoes to breed. In our study, breeding sites near houses, and having farms or houses close to swamps increased odds of malaria infection. This combined effect of exposures emphasizes the need for multiple environmental and behavioral interventions to reduce risk of malaria exposure. Malaria prevention messages to the public in this area should emphasize responsible land use practices to reduce the creation of mosquito breeding habitats in the environment [18, 19].

We identified children under five years of age as the most affected by malaria. This disproportionate burden has been widely reported both in Uganda and globally [4, 8, 16, 20]. In addition, females were twice as affected as males., a finding reported previously in multiple districts of Uganda [21]. Females in Kole District might engage in activities that increase their exposure to mosquitoes. During our study, we observed that cooking areas were outside the house, meaning that women would likely be exposed in the evenings while preparing meals. It should be noted that in comparison to males, females are also more likely to report fevers to health facilities and have more opportunities to be tested for malaria during child health care or antenatal visits [21]. However, pregnancy may also increase susceptibility [22]. In Uganda, prevalence of malaria during pregnancy was 30% in 2017, increasing the risk of maternal anemia and low birth weight babies [10]. Malaria control initiatives in this area – and likely other high-transmission areas in Uganda – should increase their targeting of pregnant women and children under five years.

Beyond the morbidity and mortality, malaria infections have negative socioeconomic implications, including treatment expenditures, lost work and school days, decreased productivity, and sometimes the loss of a household breadwinner [23]. The 2014–2020 Uganda Malaria Reduction Strategic Plan aimed to accelerate nationwide scale up of cost-effective malaria prevention and treatment interventions [24]. When scaled up, the combination of IRS, distribution of LLINs, and test-and-treat interventions contributed to a 27% reduction in the national incidence of malaria between 2017 and 2018 [8]. Ugandan researchers estimated that using a district-led approach for IRS, the overall cost per structure sprayed is UGX 28,400 (8 US$) and the average cost per person protected is UGX 7,200 (2 US$) [25]. However, costs are increased by additional measures, such as environmental compliance; a previous recent IRS in Uganda cost approximately USD 12 million to cover just 10 districts (unpublished data). In contrast, the cost of treating malaria is estimated to be between UGX 1,500 (0.41 US$) and UGX 13,800 (3.88 US$) per person per month [24]. Thus, while consistent IRS, removal of vector breeding sites, and consistent distribution and use of LLINs in the affected areas are effective, they may not be economically feasible. Community leaders can be encouraged to conduct education campaigns that raise awareness and encourage the use of LLINs and removal of stagnant water to address risk where IRS is not economically feasible.

## Limitations

During the search for cases, we did not review health records from the integrated community case management of malaria (iCCM) for children under five years in Kole District. This might have led to an underestimation of the magnitude of the outbreak among children under five years. In addition, most persons visit Health Center II (lower-level health facilities) first for malaria treatment. However, we did not visit these facilities to search for cases due to reported antimalarial stockouts during the outbreak period; instead, we visited the five health facilities where suspected cases from the lower health facilities were referred. Finally, given the high attack rates we reported in our investigation, it is possible that controls were infected but asymptomatic at the time of interview. This could have introduced a misclassification bias and underestimated our associations.

## Conclusion

Stagnant water near houses likely facilitated this outbreak through increases in mosquito breeding sites following rains. Inadequate preventive measures such as absence of IRS likely facilitated vector-human contact to enhance the outbreak. Re-introduction of IRS, re-distribution of LLIN in Kole District, and sensitizing communities about removing mosquito breeding sites might reduce the risk of future outbreaks.

### Public health actions

We sensitized the community members and leaders on malaria and easy preventive actions like removing abandoned containers around their houses that act as mosquito breeding sites and consistent use of LLIN.

## Data Availability

The datasets upon which our findings are based belong to the Uganda Public Health Fellowship Program. For confidentiality reasons the datasets are not publicly available. However, the data sets can be availed upon reasonable request from the corresponding author and with permission from the Uganda Public Health Fellowship Program.

## List of abbreviations

AR: Attack rate.
CI: Confidence interval.
DHIS2: District Health Information Software 2.
HC: Health Centre.
iCCM: integrated community case management of malaria.
IRS: Indoor residual spraying.
LLIN: Long-lasting insecticide-treated bed nets.
MoH: Uganda Ministry of Health.
NMCD: National Malaria Control Division.
OR: Odds ratio.
RDT: Rapid diagnostic test.
UBOS: Uganda Bureau of Statistics.
WHO: World Health Organization.
